# Pocketable and Smart Electrohydrodynamic Pump for Clothes

**DOI:** 10.1021/acsami.3c15274

**Published:** 2023-12-14

**Authors:** Yu Kuwajima, Yuya Yamaguchi, Yuhei Yamada, Takafumi Morita, Ardi Wiranata, Ayato Minaminosono, Naoki Hosoya, Yasuaki Kakehi, Shingo Maeda

**Affiliations:** † Department of Engineering Science and Mechanics, Shibaura Institute of Technology, 3-7-5, Toyosu, Koto-ku, Tokyo 135-8548, Japan; ‡ Living Systems Materialogy Research Group, International Research Frontiers Initiative, Tokyo Institute of Technology, 4259, Nagatsuta-Cho, Midori-Ku, Yokohama, Kanagawa 226-8501, Japan; § The University of Tokyo, 7-3-1, Hongo, Bunkyo-ku, Tokyo 113-8654, Japan; ∥ Department of Mechanical and Industrial Engineering, Universitas Gadjah Mada, Jalan Grafika No. 2, Yogyakarta 55281, Indonesia; ⊥ Department of Mechanical Engineering, Tokyo Institute of Technology, Ookayama, Meguro-ku, Tokyo 152-8550, Japan

**Keywords:** electrohydrodynamics, pump, self-sensing, wearable, thermal control

## Abstract

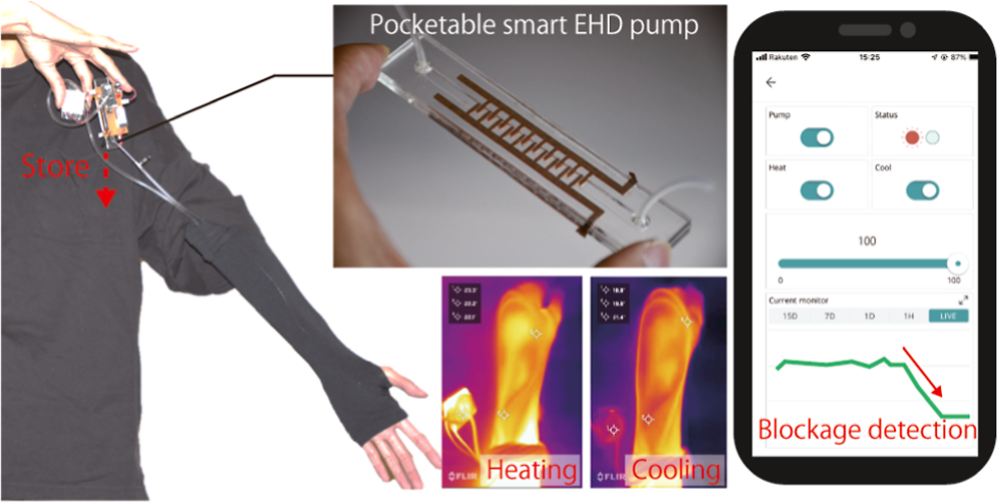

Seamlessly fusing fashion and functionality can redefine wearable technology and enhance the quality of life. We propose a pocketable and smart electrohydrodynamic pump (PSEP) with self-sensing capability for wearable thermal controls. Overcoming the constraints of traditional liquid-cooled wearables, PSEP with dimensions of 10 × 2 × 1.05 cm and a weight of 10 g is sufficiently compact to fit into a shirt pocket, providing stylish and unobtrusive thermal control. Silent operation coupled with the unique self-sensing ability to monitor the flow rate ensures system reliability without cumbersome additional components. The significant contribution of our study is the formulation and validation of a theoretical model for self-sensing in EHD pumps, thereby introducing an innovative functionality to EHD pump technology. PSEP can deliver temperature changes of up to 3 °C, considerably improving personal comfort. Additionally, the PSEP system features an intuitive, smartphone-compatible interface for seamless wireless control and monitoring, enhancing user interaction and convenience. Furthermore, the ability to detect and notify users of flow blockages, achieved by self-sensing, ensures an efficient and long-term operation. Through its blend of compact design, intelligent functionality, and stylish integration into daily wear, PSEP reshapes the landscape of wearable thermal control technology and offers a promising avenue for enhancing personal comfort in daily life.

## Introduction

**Figure 1:**
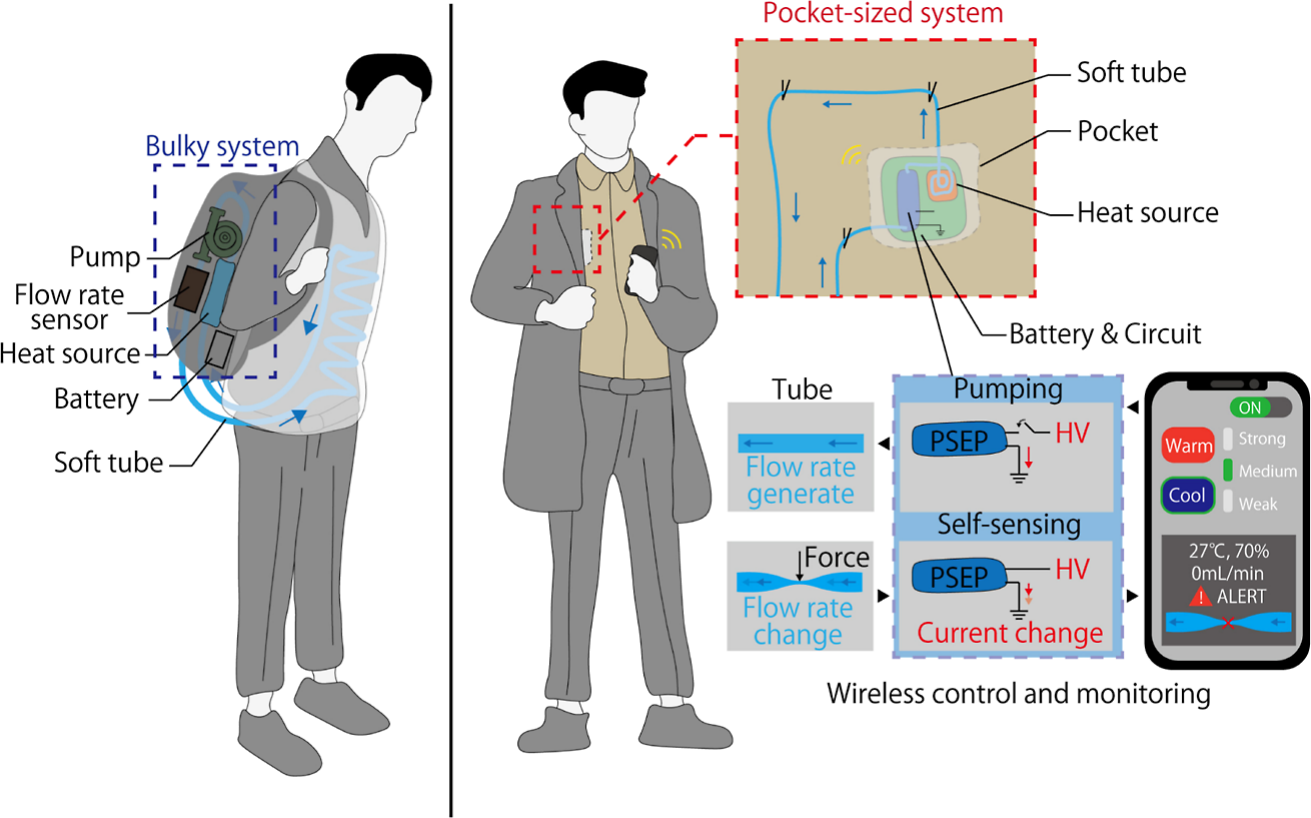
Advanced liquid cooling systems: from conventional to PSEP-enabled clothing. Left side: conventional liquid cooling clothing. The main elements are bloated, limiting the application area. Right side: proposed system employs a PSEP. The main elements are compact to be stored in clothing and do not limit the clothing style and area of application. PSEP’s self-sensing capability of flow rate improves system reliability and also keeps the system compact, as there is no need for additional sensors and associated power supply. In addition, the user can control and monitor the system via a smartphone while stored. The PSEP can detect flow obstructions and inform the user via a smartphone, ensuring reliability during use.

Wearable thermal control devices have various applications, such as enhancing individual thermal comfort,^1^ providing thermal feedback in virtual and augmented reality spaces,^2^ serving as thermal camouflage against infrared detection,^3^ and offering thermotherapy for health issues.^4^ Researchers have investigated effective wearable thermal control devices from material, design, and system perspectives, employing air transport,^5^ liquid transport,^6^ thermoelectric elements,^7^ phase-change materials,^8^ and textiles.^9^ Soft robotics,^10−14^ which has attracted a lot of attention in recent years, also offers a unique opportunity to make wearable devices that are more adaptable, comfortable, and closely conform to the human body. Due to their high cooling capabilities, liquid transport systems have been commonly used for cooling^15^ since their initial proposal in 1959.^16^ Liquid-cooling garments have been developed for specialized users such as racing car drivers, surgeons, chemotherapy and multiple sclerosis patients, athletes, and hazardous material handlers.^17^ In these devices, chilled or warmed liquids circulate through tubes via a pump, facilitating heat exchange with the human body. Other examples include liquid transport for wearable tactile presentation via chemical reactions^18^ and weight control,^19^ expanding the application range. However, pumps for liquid transport generate significant noise and weight, and their bulky systems make them cumbersome to wear, posing challenges for casual use (Figure 1, left side). Furthermore, their standardized design limits user choice and fails to cater to individual needs.

Recently, small pumps have been developed based on various driving principles. Figure S1 and Table S1 show their sizes, maximum flow rates, driving mechanisms, and applications.^20−32^ In particular, electrohydrodynamic (EHD) pumps, which operate silently and do not generate heat, are gaining attention as driving sources for wearable thermal control devices. EHD pumps produce a continuous silent flow in the working fluid as the charge injected by the electrodes moves according to the electric field.^33^ Compared with other pumps, EHD pumps have a high maximum flow rate, determining the rate of heat transfer, making them suitable for thermal control devices. Recent studies have proposed EHD pumps made of stretchable materials,^20^ modular designs for easy expansion,^34^ and shaped fibers for integration with textiles.^21^

In wearable thermal control devices using liquid transport systems, soft tubes are preferred for their affinity with humans. Soft tubes have the advantage of not interfering with human movement. In unmaintained conditions, such as in human living spaces, physical contact with human motion and the external environment can cause tube deformation, potentially leading to significant issues such as downtime, reduced operating efficiency, and failures caused by localized pressure concentrations. Therefore, real-time flow rate sensing is required to detect blockages in the flow path. Adding a flow rate sensor to the system may require an additional power supply system, increasing the bulk and complexity of the system. Researchers have effectively demonstrated self-sensing actuators as an approach for system miniaturization and multifunctionality.^35−39^ The realization of EHD pumps with self-sensing functionalities can lead to innovative smart pumps. Despite this, there have been few attempts to realize self-sensing pumps. Although, researchers have developed self-sensing piezoelectric pumps using piezo actuators.^40^ The driving mechanism of a piezo pump uses mechanical vibration, which produces noise. A better approach for pumps in wearable systems is to implement silent operations such as EHD pumps.

Recent research has investigated the response of the current to the flow rate flowing externally into the EHD pump and its sensing mechanism.^41^ However, this approach alone is insufficient in the context of wearable devices, where real-time self-sensing of flow rates is essential. It is necessary to establish a model equation to check the response to self-sensing flow rates and to validate the behavior based on this equation. This is the core of our approach in the development of smart EHD pumps with a self-sensing functionality.

Herein, we propose a liquid transport system with a pocketable and smart EHD-driven pump (PSEP) (Figure 1, right side). Unlike conventional liquid transport systems, PSEP silently transports the working liquid without additional components such as a power supply system or inside the pump for its self-sensing ability; thus, the entire system is simple, compact, and lightweight. Our system is portable and easy to wear and take off; thus, it does not restrict the user’s range of movement or clothing design. First, we demonstrate the current response of PSEP to the flow rate of the loaded tube. Subsequently, we validated the PSEP model and evaluated its performance and reliability. Furthermore, we developed a PSEP-driven liquid transport system for wearable thermal control. Users can store the main elements, such as the PSEP, power/control circuit, and heat source, in a pocket and perform various functions such as heating, cooling, and flow blockage detection through a smartphone. Moreover, the self-sensing of PSEP promotes efficient and reliable operation through feedback from the user. PSEP supports wearable thermal control devices by solving the problems of noise, weight, and large size and contributes to the next generation of wearable thermal control devices designed to meet individual needs.

## Results and Discussion

### PSEP Concept

**Figure 2:**
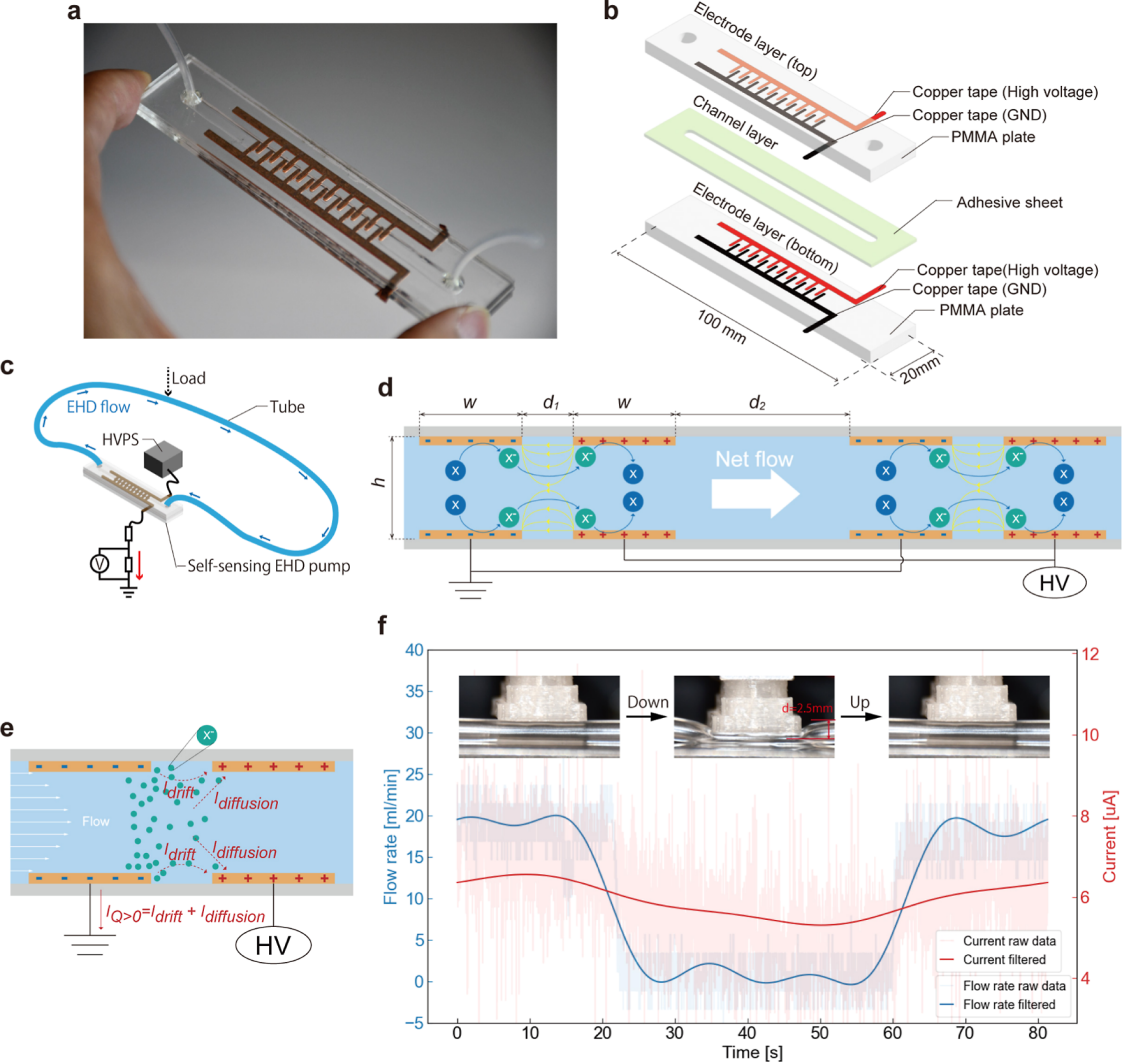
Design, mechanisms, and operation of PSEP. (a) Photograph of PSEP and (b) its structure and materials. (c) PSEP circulates the liquid using a high voltage input and self-senses the flow rate according to current. (d) EHD-driven pumping mechanism; ions X^–^ injected from the negative electrodes move according to the lines of electric force (yellow lines) and generate a flow. Multiple electrode pairs within the interdigitated electrode further accelerate this flow. (e) In the presence of a flow rate, the EHD pump current is the sum of the drift current, which follows Ohm’s law, and the diffusion current due to the diffusion of ions generated by the flow. (f) PSEP produced a flow at 3 kV; the current decreases when the flow is obstructed by tube deformation.

Figure 2a shows the developed PSEP, of size 21 cm^3^ (10 × 2 × 1.05 cm) and weight 10 g. The PSEP design is based on conventional EHD pumps comprising three main components: electrodes, flow channels, and an insulating fluid. The charges injected from the electrodes into the insulating fluid move along the electric field, generating a unidirectional flow. As shown in Figure 2b, the PSEP comprises two electrode layers with interdigitated electrode structures and a flow channel layer created using a single adhesive sheet. Each interdigitated electrode structure contains 10 pairs of electrodes, accelerating the EHD flow and producing an increased output.^42^ Moreover, these electrodes simplify expansion and fabrication, integrate wiring, and reduce fluid friction. The straightforward design and use of accessible and easily processable materials enable rapid and cost-effective production. The PSEP transports liquids by applying kV-order voltage and estimates the flow rate based on the change in current, as shown in Figure 2c. Figure 2d illustrates the EHD pumping mechanism of the PSEP. In our PSEP, ions are injected into the working fluid from the electrode under the influence of a strong electric field. When the applied electric field reaches critical strength, ions can overcome the energy barrier and tunnel directly from the cathode’s surface into the working fluid. Injection in insulating fluids containing many electronegative molecules, such as fluorine-based substances, is more likely to occur at the negative electrode because of its lower energy barrier compared to the positive electrode. The Coulomb force pushes the injected ions along the electric field lines. During this process, ions repeatedly collide with the fluid molecules. Consequently, a flow is generated from the negative to the positive electrode. Figure 2e shows the self-sensing mechanism of the PSEP flow rate. A current of the order of μA is generated in the PSEP, and the output flow rate can be estimated from the current. Therefore, it has the advantage of keeping the system lightweight and compact without additional flow rate sensors and power supplies. In our previous work,^41^ we reported the development of a hydraulically driven suction cup with contact detection that uses an EHD pump and we found the relation between flow rate, *Q*, and electrical current between electrodes, *I*, as
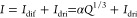
1where *I*_dri_ is the drift current which follows Ohm’s law, *I*_dif_ is the diffusion current arising from the concentration gradient of ions due to the flow of liquid,^43^ and α is a constant. We assume that the same relationship holds for the flow rate of our self-sensing device. α is constant and is derived from previous studies of reactor model equations. This constant is determined by the ion concentration and the geometrical parameters of the electrodes and flow paths. This study treats α which is a coefficient of one-third the power of the flow rate as sensitivity. Although we ignored the effect of the EHD pump’s instability in our previous study, it becomes significant when we use the device for a long time. Careful selection of the liquid and electrode materials has a high potential for solving instability. Here, we propose a simple data processing method to exclude instability. We assumed that the main cause of instability was the working fluid, whose chemical composition gradually changed over time. Since the electrical conductivity of the fluid changes, the drift current rather than the diffusion current fluctuates: *I*(*t*) = *I*_dif_(*Q*) + *I*_dri_(*t*). To eliminate time dependence, we define a reference time, *t*′, and calculate the current variation
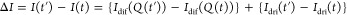
2which we use as an evaluation parameter. Assuming that the instability effect is much slower than the change of *Q* and *t*′ – *t* is sufficiently small, the terms in the second curved bracket can be ignored. *t*′ can be considered as the serial reference times defined at certain intervals. In this case, the interval should be longer than the dynamics of the flow rate’s change that must be detected and be shorter than those of the instability dynamics. Therefore, in this study, we define *t*′ as the time at which *I*(*t*′) becomes the maximum in the interval for the following reason. Because the flow in the EHD system is sensitive to the environment, *I* fluctuates even when the setting is constant. Because the fluctuation is caused by hydrodynamic dissipation, *I* is maximized when steady flow is ideally realized. Therefore, it is reasonable to adopt *I*_max_ = *I*(*t*′) as the quantity characterizing the steady state without a load. Then, we obtain
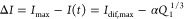
3where *I*_dif,max_ denotes the maximum diffusion current without any fluctuations or loads. Equation 3 shows the calibration curve of the PSEP for detecting the flow rate by the change in current. In this study, we constructed a closed system in which the liquid is circulated through the PSEP, as shown in Figure 2c. We demonstrated self-sensing functionality and investigated the relationship between the flow rate and current in the PSEP. An applied voltage of 3 kV to the PSEP causes EHD pumping and circulation of the working fluid. The experimental results in Figure 2f show a flow rate of 20 mL/min and a current of 7 μA. When a 25 N load was applied to deform the tube *d* = 2.5 mm for 40 s, the flow rate dropped to 0 mL/min, and the current decreased by a maximum of 1.5 μA. When the load was removed, the current increased by 1.5 μA, returning to the preload input state. These experimental results suggest that the change in current is due to the theoretically estimated flow rate-dependent diffusion current, demonstrating PSEP’s self-sensing through current monitoring.

### Validation of the Model Equation for Self-Sensing

**Figure 3:**
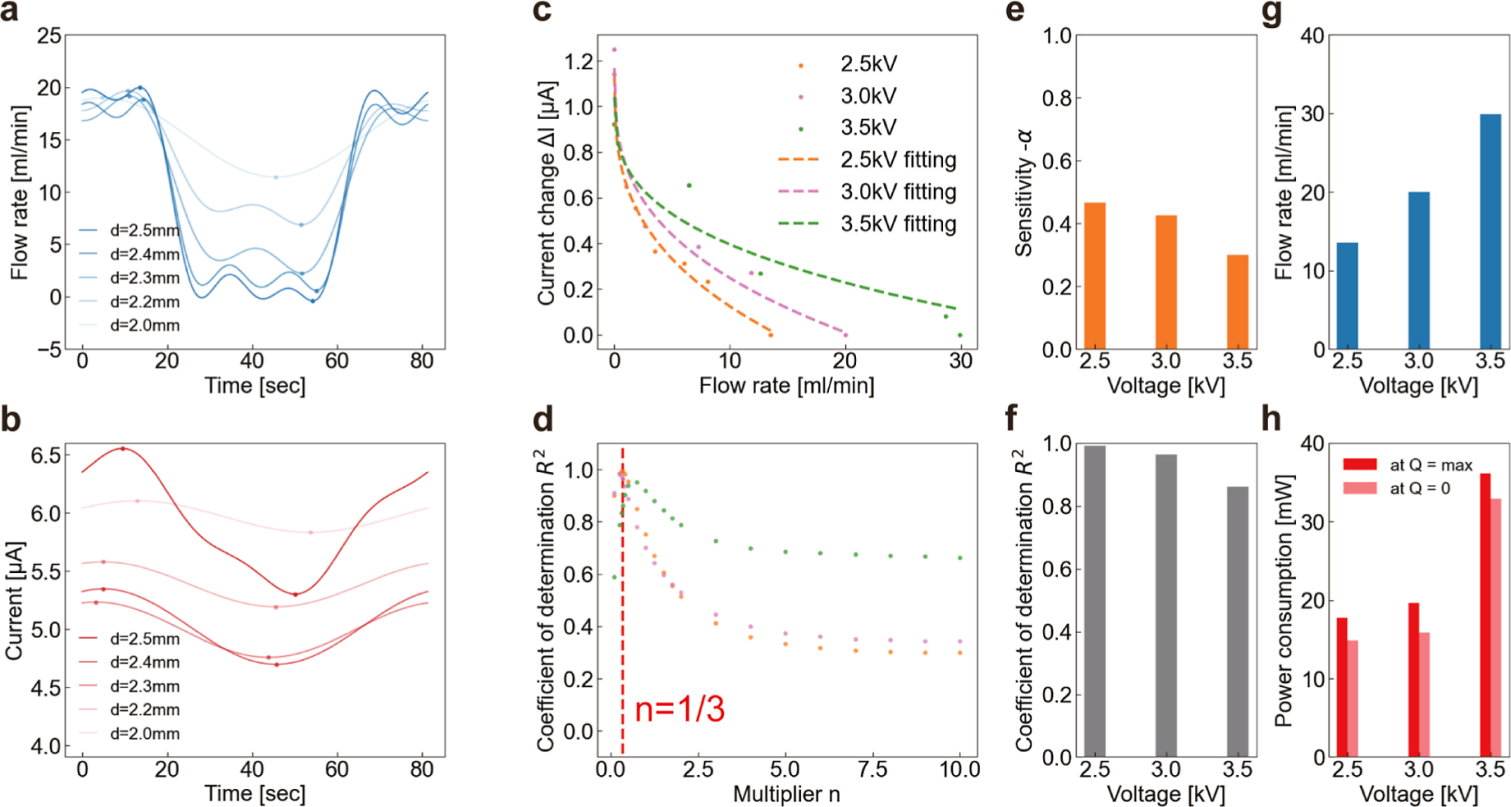
Validation and performance evaluations. Time variation of (a) flow rate and (b) current during tube deformation. The voltage of 3 kV was applied, and five patterns of tube deformation *d* = 2.5, 2.4, 2.3, 2.2, and 2.0 mm were tested. (c) Flow rate vs change in the current at voltages of 2.5, 3.0, and 3.5 kV. Experimental values (dots) and theoretical fitting (dotted lines) were plotted. (d) Peaks of *R*-squared exist at a point close to the theoretical value of the multiplier 1/3. (e) Sensitivity α and (f) coefficient of determination *R*^2^ are plotted, respectively. (g) The maximum flow rate and (h) power consumption increase with applied voltage; power consumption is self-saving when the flow rate is zero.

We investigated the relationship between the PSEP flow rate and current to validate the model equation. We varied the flow rate and current by controlling the tube’s deformation, *d* = 2.0–2.5 mm. Figure 3a,b shows the time evolution of the flow rate and current of the PSEP under the 3.0 kV. The two dots in each curve indicate the maximum and minimum values of *Q* and *I*, denoted as *Q*_max_, *Q*_min_, *I*_max_ and *I*_min_. As expected, in the initial 0 to 20 s period, before deformation was applied to the tube, fluctuations in current were observed despite only slight changes in flow rate. Moreover, we observed that the greater the change in flow rate, the greater the change in current. Figure 3c shows the relationship between the change in current (Δ*I* = *I*_max_ – *I*(*t*)) and the flow rate when a load is imposed (*Q*_min_) at each applied voltage. The data was fitted for each applied voltage using eq 3. The fittings are better for *V* = 2.5 and 3.0 kV, suggesting that imposing a higher voltage induces instability and is unsuitable for the sensor. To verify the validity of eq 3, we performed fitting using
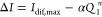
4where *n* is a variable. We compared the coefficient of determination *R*^2^ with varying *n*. Here, *R*^2^ indicates the goodness of fit; as *R*^2^ is closer to 1, the better the model equation fits our experimental data. Figure 3d shows these results. The peak occurs at approximately *n* = 1/3 (red dashed lines), indicating the validity of this model equation.

### Performances of PSEP

**Figure 4:**
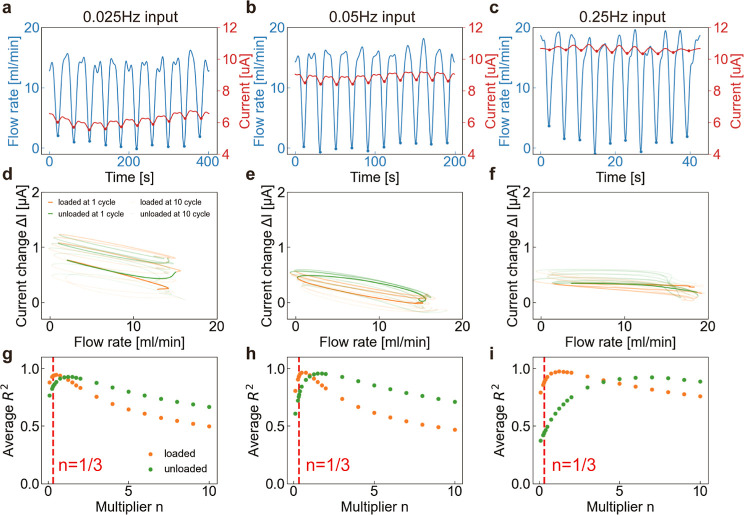
Cyclic testing to evaluate self-sensing performance reliability. Time trends of current and flow rate were measured when 10 cycles of loading were applied to the tube at frequencies of (a) 0.025 Hz, (b) 0.05 Hz, and (c) 0.25 Hz. (d–f) Flow rate vs the change in current and (g–i) multiplier vs average *R*^2^ of 10 cycles with load (orange line) and without load (green line) at each input frequency.

We evaluated the PSEP performance at each applied voltage. We estimated α from the experimental results obtained by applying least-squares fitting. In Figure 3c, eq 4 is drawn with the estimated α as a dashed line. α which corresponds to the sensitivity and coefficient of determination *R*^2^ increases as the applied voltage decreases, as shown in Figure 3e,f. In contrast, the maximum flow rate increases with an increase in applied voltage, as shown in Figure 3g. The maximum flow rate is 30 mL/min at 3.5 kV voltage. However, at 3.5 kV, we observed the dielectric breakdown during the experiment, making it unsuitable for long-term use. Therefore, 3.0 kV or lower applied voltage was set for sensing and pumping and to ensure stability. Furthermore, as shown in Figure 3h, PSEP saves energy when the flow rate *Q* = 0 compared to when the flow rate is maximum. To investigate the reliability and cyclic performance of the self-sensing performance, we conducted 10-cycle tests at a voltage of 3.0 kV; the tube was deformed cyclically in three patterns at 0.25, 0.05, and 0.025 Hz. Figure 4a–c shows the time evolution of the flow rate and current at each input frequency. At all input frequencies, the current responded to changes in the flow rate. Figure 4d–f shows the relationship between the flow rate and the change in current at each input frequency. Here, we use *I*_max_ to define Δ*I* as the maximum current before the start of the cycle. We categorized the plots into loading (orange) and unloading (green) processes. We observed hysteresis and fluctuations in the current at all input frequencies. Figure 4g–i shows theoretical verification similar to that in Figure 3d. Here, the average *R*^2^ over all the cycles, distinguishing the loading and unloading processes were evaluated. First, we focused on the input frequency. At lower frequencies, the change in the current to flow rate was large and the average *R*^2^ peaked at approximately *n* = 1/3. However, the change in the current tended to dissipate from cycle to cycle. We attribute this to the observed dynamics of instability caused by long measurement times rather than frequency effects. This result indicates the limitation of continuous measurement intervals and the need for a redefinition of the reference time *t*′. However, at high frequencies, the ratio of the change in the current to the flow rate was small, and the peak of the average *R*^2^ deviated from *n* = 1/3, probably because the next input started before the current relaxation. In addition, we derived eq 3 as the change in flow from the steady state; this assumption breaks at high frequencies. Therefore, the flow rate can be properly estimated up to 0.05 Hz. As a reference, we have calculated the response time (*t*_2_ – *t*_1_) as the difference between the time of the lower peak of the flow rate (*t*_1_) and that of current (*t*_2_) at 0.05 Hz, and the response time within 2 s (Figure S7). Second, we focus on the loading and unloading processes. Compared with loading, the unloading process increased the average *R*^2^ peak and deviated from *n* = 1/3. Loading and unloading are unsteady processes that change the channel width; however, loading reduces the Reynolds number, whereas unloading increases it. Since our theoretical equation is derived assuming steady-state conditions, the approximation is more likely to hold for operations that lower the Reynolds number compared to operations that increase it. The result indicates that the self-sensing of the PSEP was more accurately measured by loading than by unloading.

### PSEP for Wearable Thermal Control Application

**Figure 5:**
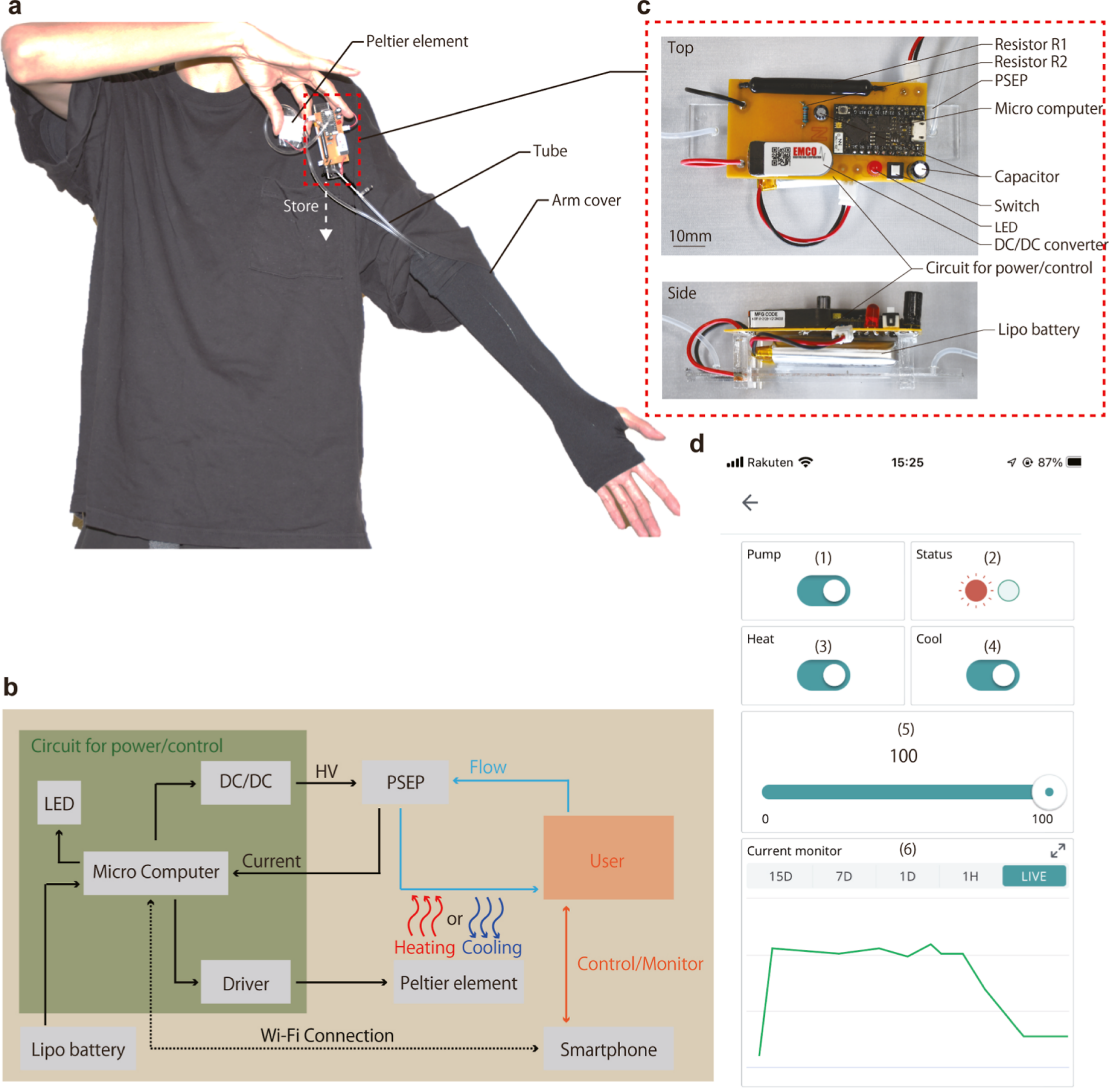
Pocket-sized thermal control system using PSEP: system architecture and user interface design (a) small and lightweight PSEP, heat source, and the power supply/control circuit are stored in the T-shirt pocket. A tube filled with working fluid was placed freely inside the arm cover for UV protection. (b) System schematic. Developed (c) power/control circuits and (d) GUI on the smartphone. The system supplies PSEP with a voltage of 2.5 kV to transport liquids cooled or heated by Peltier elements. The system also constantly monitors the current and detects tube blockage. Users can operate the cooling and heating systems and check the operation status from their smartphones.

**Figure 6:**
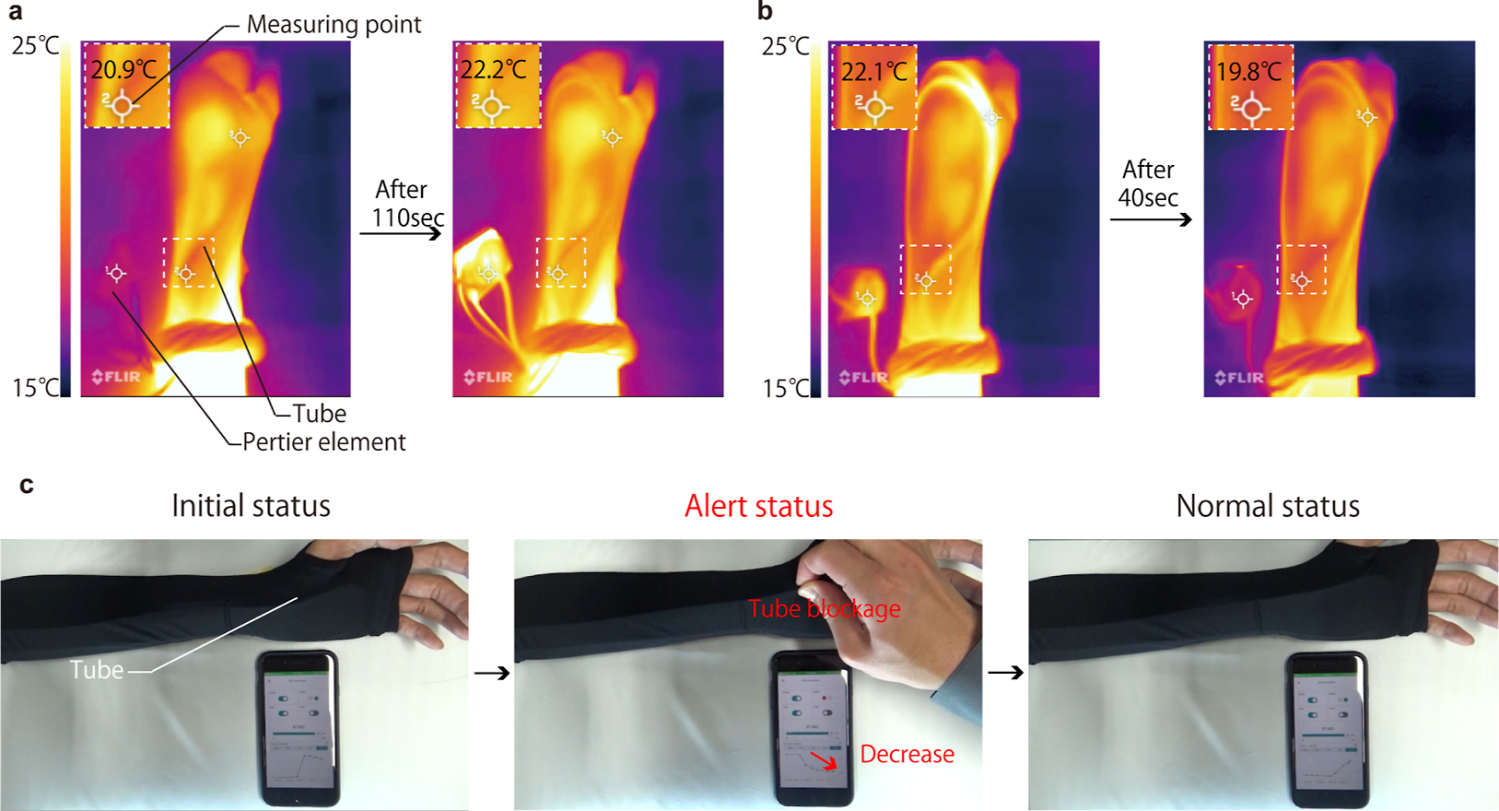
Demonstrations of pocket-sized thermal control system functions. (a) Heating and (b) cooling functions. The working liquid heated and cooled by the Peltier element is transported by the PSEP to control the temperature of the tubes placed in the arm. The colors in the thermal image correspond to the top left color bar. The thermal image on the left shows the initial state. The right infrared image shows 110 and 40 s after switching on the Peltier element and PSEP. A voltage of 3.7 and −3.7 V are applied to the Peltier element during heating and cooling, respectively. The temperature changes at the measurement point were observed. (c) Flow blockage function. In this demonstration, the tube is deliberately pinched to simulate a blockage in the flow path. GUI on the smartphone; the status is abnormal (red) when the tube is pinched and returns to normal (green) when the tube is unpinched. The current is monitored simultaneously.

We designed a pocketable system to regulate liquid circulation employing PSEP, targeting wearable thermal control applications. The primary constituents of the system, as shown in Figure 5a, encode the PSEP, heat source, and circuit for the control and power supply. The entire system fits snugly in a T-shirt pocket with dimensions of 10 × 10 cm and weighs a mere 30 g including the tubes and working liquid. Incorporating the tube within an arm cover protects against ultraviolet while fostering thermal comfort for the user. A schematic of the system, provided in Figure 5b, elucidates the transportation process of the chilled or warmed working fluid by PSEP, facilitated by a voltage of 2.5 kV derived from the circuit portrayed in Figure 5c. After the heat exchange with an individual through the tubes, the working fluid alters the temperature of the target area before being cooled or heated. In the operation of our PSEP, minimal heat generation is expected. The heat can be estimated from the input energy, calculated as the product of current and voltage. For instance, as illustrated in Figure 3b, with a voltage of 3 kV and a current of 6.5 μA, the input energy is calculated to be 19.5 mW, indicating that significant heating of the PSEP is unlikely. Indeed, no notable temperature changes were observed in previous studies of EHD pumps. This characteristic of low heat generation makes the PSEP suitable for applications that require both heating and cooling capabilities. The closed-loop design of the system and the principle of PSEP’s pumping make it less susceptible to mechanical disturbances. Also, the self-sensing feature enhances the reliability of heat transport. The system can detect excessive load on the tube, construing such an event as inability to transport fluid, and accordingly issue a warning to the user. Connecting to a smartphone via Wi-Fi is another important feature. This enables the user to regulate and manage the system while it is stored in a pocket. Figure 5d shows an intuitive graphical user interface (GUI) operating on a smartphone designed to facilitate user interaction. We observed changes in temperature by using an infrared camera (FLIER ONE Pro, FLIR). The temperature variations during heating and cooling were Δ*T*_heat_ = 22.2–20.9 = 1.3 °C and Δ*T*_cool_ = 19.8–22.1 = −2.3 °C, as shown in Figure 6a,b, respectively. Figure S9 also shows the temperature change versus time for each process. We demonstrated its effectiveness as a thermal control device. Movies S1 and S2 show the heating and cooling operations, respectively. Figure 6c shows the detection of blockages. We deliberately blocked the tube and monitored it from the smartphone GUI. Movie S3 shows the sequence of operation including device operation. Thus, PSEP innovations featuring quiet liquid transport and self-sensing capabilities offer high reliability and will allow users to incorporate thermal control features into their garments.

## Experimental Section

### Working Liquid

The physical properties of the working fluid, (fluorinated liquid) (Novec 7300, 3M), are as follows. EHD pumping requires low conductivity (<10^–7^ S/m), high dielectric withstand voltage, and high dielectric constant. Novec 7300 has a conductivity of 10^–9^ S/m, a dielectric withstand voltage of 5–6 kV (gap: 0.5 mm), and a dielectric constant of 6.1. It also has a boiling point of 76 °C, high thermal conductivity, ultralow toxicity, zero ozone depletion potential, zero flash point, and nonflammability, making it suitable for wearable thermal control devices.

### Flow State Estimation in PSEP for Theoretical Validation

PSEP has a channel height of 0.5 mm, a channel width of 5 mm, a channel cross-sectional area of 0.025 mm^2^, and a hydraulic diameter of 0.910 mm. Reynolds number *Re* = 714 < 2000 at the maximum flow velocity of 0.549 m/s (30 mL/min) at a voltage of 3.0 kV. Therefore, the flow investigated in this study was within the laminar flow range for which the model equation was valid.

### GUI on Smartphone

The GUI of the proposed wearable device is intuitive and runs on a user’s smartphone. This GUI is implemented using the Arduino IOT remote IoT platform. Figure 4D shows the screen of the GUI on the smartphone, which has five functions. (1): Turn on/off the pumping function of PSEP. (2): Lights up red in the case of blockage and green if there is no problem with the operation. The change in current of 0.3 μA was used as the threshold. Turns on/off the (3): cooling (4): heating function of the Peltier element. (5): The output power of the Peltier element is also adjustable by the slider. The number indicates the percentage of the absolute value of the voltage applied to the Peltier element. (6): Current values are monitored in real-time, and irregular inputs can be checked.

The GUI of the proposed wearable device is intuitive and runs on the user’s smartphone. Figure 4D shows the GUI screen on a smartphone. The GUI was implemented using an Arduino IOT remote IoT platform. GUI has five functions. (1): On/off PSEP pumping function. (2): Indicate blockage. The red light indicates blockage, and green light indicates no problem with the operation; the change in current of 0.3 μA was used as the threshold. Three and (4): On/off the cooling and heating function of the Peltier element. (5): The output power of the Peltier element was adjusted by the slider. The number indicates the percentage of the absolute value of the voltage applied to the Peltier element. (6): Monitored current values in real time and checked with irregular inputs.

## Conclusions

This study theoretically examines the relationship between the flow and current in EHD pumps and introduces PSEP, which is based on the conventional EHD pump design and in this study, the relationship between flow rate and current, which is the output of an EHD pump, is investigated theoretically and experimentally, and a PSEP with pumping and flow rate self-detection functions is introduced from a conventional EHD pump design. We experimentally confirmed its response, and the proposed theory eliminated the current instability. Theoretical verification and performance evaluation showed that the flow rate multiplier was equal to 1/3 as indicated by the theoretical equation, and the coefficient of determination was above 0.9, indicating the consistency of the model. Pressure and flow rate as pumping performance and sensitivity, accuracy, and responsiveness as self-sensing performance were comprehensively evaluated. Finally, we present a breakthrough in wearable thermal control technology, namely, a lightweight and compact liquid circulation system powered by PSEP. Our system fits into the 10 × 10 cm T-shirt pocket and weighs only 100 g. Our system facilitates personal thermal management by providing cooling and heating functions, effectively combining fashion and functionality. Moreover, Wi-Fi connectivity allows remote control and expands the operability of the user. Emphatically, the self-sensing function provides critical feedback on flow blockages, enhancing the system’s reliability. This significant integration of practicality and style in wearable thermal control paves the way for the future of personalized thermal control. In future research, we will first focus on improving the materials and shapes of electrodes for long-term and heightened sensing accuracy. We will also conduct long-term cycle and continuous operation tests to validate the reliability of our PSEP. Subsequent to this, we plan on integrating multiple miniaturized PSEP arrays, precisely engineered to identify malfunctions and relay real-time feedback to users. Drawing from pioneering studies that illustrated the use of self-healing liquids transported by EHD pumps,^44^ our vision encompasses channeling these innovative liquids to the malfunction points pinpointed by PSEP, fortifying the system’s resilience. The proposed PSEP has the potential to become a new generation of universally applicable wearable cooling and heating devices because of its multifunctionality, silent operation, and lightweight.

## Supporting Information Available

Plot of various pump sizes and flow rates; pump operating principle, maximum flow rate, size, and application are summarized; electrode design of PSEP; digital and rapid fabrication process of electrode, substrate, and channel layers; experimental setup; compression test to clarify the relationship of tube deformation to the load applied to the tube; FFT analysis of currents and flow rates for cycling tests; the response time (*t_2_-t_1_*) as the difference between the time of the lower peak of the flow rate (*t*_1_) and that of current (*t_2_*) at 0.05 Hz input; electrical schematic of the power/control circuit; and temperature versus time in heating (left) and cooling (right) demonstrations (PDF)
Heating function of wearable application (MP4)
Cooling function of wearable application (MP4)
Blockage detection, wireless control, and monitoring functions of wearable application (MP4)
