# Supplementary material for: Pocketable and Smart Electrohydrodynamic Pump for Clothes
Source: ACS Appl Mater Interfaces. 2023 Dec 14;16(1):1883–91. doi: 10.1021/acsami.3c15274 (PMC10788827; doi:10.1021/acsami.3c15274)
Supplement: Supplementary file 1 — am3c15274_si_001.pdf [file am3c15274_si_001.pdf]

Supporting Information

Pocketable and Smart Electrohydrodynamic Pump for Clothes

Yu Kuwajima[1], Yuya Yamaguchi[1], Yuhei Yamada[2], Takafumi Morita[3], Ardi Wiranata[4], Ayato Minaminosono[1], Naoki Hosoya[1], Yasuaki Kakehi[3] and Shingo Maeda*[5]*

[1] Department of Engineering Science and Mechanics, Shibaura Institute of Technology, 3-7-5, Toyosu, Koto-ku, Tokyo, 135-8548, Japan

[2] Living Systems Materialogy Research Group, International Research Frontiers Initiative, Tokyo Institute of Technology, 4259, Nagatsuta-Cho, Midori-Ku, Yokohama, Kanagawa, 226-8501, Japan

[3] The University of Tokyo, 7-3-1, Hongo, Bunkyo-ku, Tokyo, 113-8654, Japan

[4] Department of Mechanical and Industrial Engineering, Universitas Gadjah Mada, Jalan Grafika No. 2, Yogyakarta, 55281, Indonesia

[5] Department of Mechanical Engineering, Tokyo Institute of Technology, Ookayama, Meguro-ku, Tokyo, 152-8550, Japan

Email: nb21105@shibaura-it.ac.jp (Yu Kuwajima), maeda.s.ao@m.titech.ac.jp (Shingo Maeda)

Materials and Methods

Design of electrode layers for PSEP

Figure S1 shows the interdigitated PSEP electrode design. We set the electrode width and gap to 0.5 mm, electrode pair gap to 1.7 mm, and the number of electrode pairs to 10. The optimal values of the relationship between the electrode and the electrode pair gaps identified in previous studies were adopted. The electrode tips were chamfered to prevent unnecessary electric field concentrations. The substrate size was 10 × 2 cm, and

the electrodes were placed 8 mm outward for wiring. We used copper tape as the electrode material and PMMA as the substrate for easy fabrication and cost-effectiveness. The copper tape and PMMA thicknesses were 0.035 and 5 mm, respectively.

Fabrication of PSEP

Figure S2 shows the digital fabrication method for PSEP. First, interdigitated shapes were cut from the copper tape using a cutting plotter (CE6000-40 plus, GRAPHTEC). Next, the interdigitated electrodes were attached to the PMMA for the electrode layer, which was cut using a laser-cutting machine (Speedy 100, Trotech). A double-sided adhesive acrylic elastomer for the channel layer was cut using a laser-cutting machine (Speedy 100, Trotech). Finally, the PSEP was fabricated by laminating two electrode layers and one channel layer. This digital fabrication process allows a high degree of design freedom and speed for mass production.

Experimental Setup

Figure S3 shows a schematic of the experimental evaluation system used in this study. The channel was filled with a working liquid before the experiment to prevent insulation breakdown owing to bubbles. HVPS (HEOPT-20B10, MATSUSADA Precision) was used to power the PSEP and induce EHD flow in the tube. The liquid was circulated through a tube with outer and inner diameters of 4 and 2 mm, respectively. The flow rate was measured using a noncontact flow rate sensor (FD-XS8, KEYENCE). Two circuit protection resistors of 100 kΩ and a shunt resistor of 5.1 kΩ

were placed in series on the GND side of the PSEP, and the current was detected from the voltage across the shunt resistor. The flow rate was varied by deforming the tube and squeezing the cross-sectional area of the flow path using a linear stage developed in previous studies to evaluate soft materials.

Compression test of soft tube

Figure S4 shows the compression test results of a tube filled with the working fluid and 3 kV applied to the PSEP. The force during compression was measured using a load cell attached to a linear stage, and each deformation force was plotted, revealing a linear relationship. Moreover, the results of Figure S4 and 2A revealed that the tube closed when a force of approximately 25 N was applied.

FFT analysis and Noise filtering

From the experimental results of the cyclic tests (Figure 4A, B, and C), FFT analysis was performed. Figure S5 shows the results of the FFT analysis for each input frequency (0.25, 0.05, and 0.025 Hz) of the tube deformation. The peaks with the highest flow rates and current amplitudes were plotted. The frequencies at which the flow rate and current peaked were 0.234, 0.050, and 0.025 Hz, confirming the self-sensing response. As the raw values of the current through the PSEP contain noise, the current data with amplitude < 0.08 μA and frequency > 0.5 Hz were filtered for evaluation.

Circuit for power/control

Figure 5C shows the developed circuit for power and control in wearable applications. The size of this circuit is 70 × 35 × 15 mm, close to that of wearable and mobile devices

and can be stored in a pocket. Figure S6 shows the circuit design. This circuit comprises a high-voltage output to the PSEP, voltage output to the Peltier element, current measurement of the PSEP, alert to the wearer in case of flow blockage, and a wireless control/monitoring function. The high-voltage output function for the EHD pump drive uses a DC/DC converter (A30P-5, XP Power). A maximum voltage output of 3kV is possible using microcontroller voltage control. A motor driver (BD6211F-E2, ROHM) applies the voltage output function to the Peltier element. The voltage applied to the Peltier element can be controlled in the range of -3.7 to 3.7 V using a PWM signal from the microcontroller, thus achieving cooling and heating functions. The voltage across the shunt resistor was read by the microcontroller to calculate the current flowing in the PSEP. A circuit protection resistor with a maximum voltage of 10 kV protects the microcontroller in case of an EHD pump breakdown. A red LED is turned on to alert the wearer. In this circuit, the microcontroller TinyPico (Unexpected Maker) was used to control the system. The microcontroller processor was an ESP32 PICO-D4 (ESP32 PICO-D4, Espressif) controlled in a general-purpose Arduino environment. The system was powered using a 1-cell lithium polymer battery. The entire system is powered by connecting a 3.7 V battery to the VBAT Pin of the TinyPico. The wireless control/monitoring function uses 2.4 GHz Wi-Fi in the ESP32 PICO-D4. Wireless communication over Wi-Fi allows users to use the device while storing it in a pocket. Furthermore, the measured data can be stored in the cloud and operated and monitored remotely.

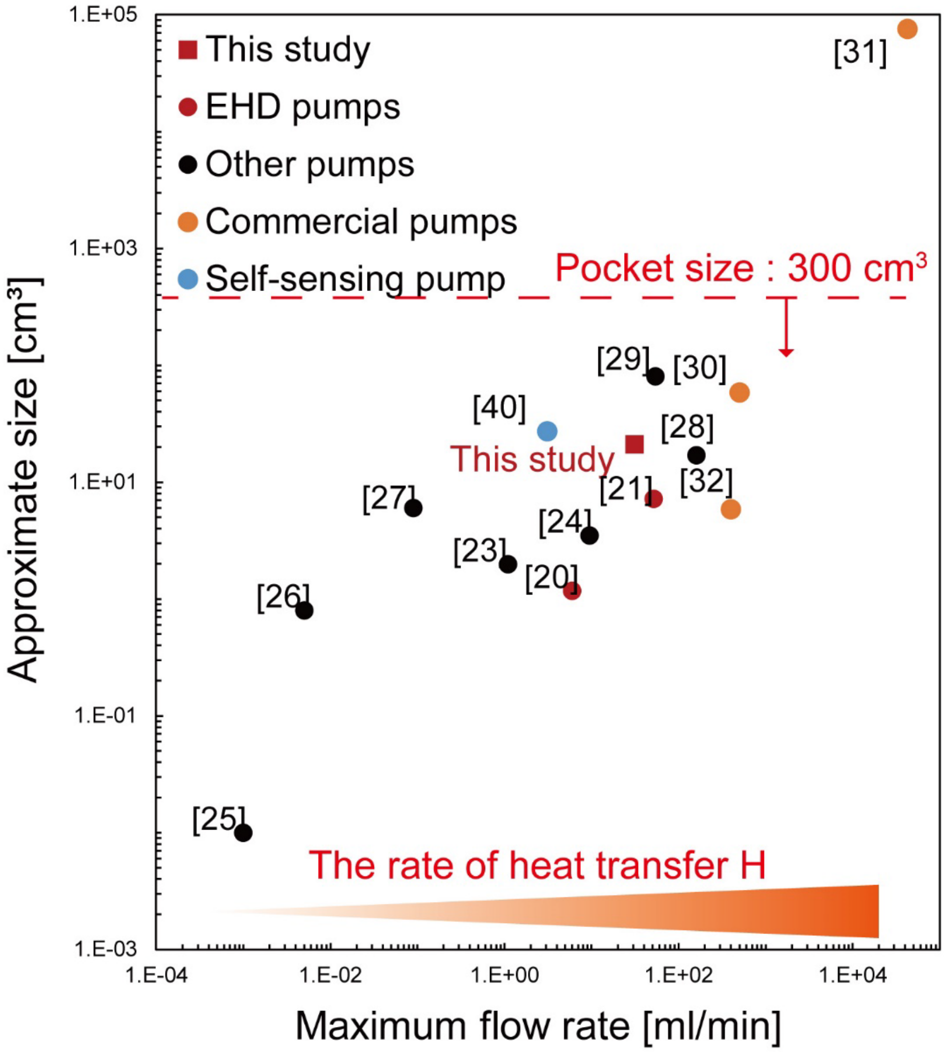

Figure S1. Plot of various pump sizes and flow rates. Roughly 300 cm^3 (15×8×2.5cm) is estimated as a pocketable size, and the PSEP is below this threshold. It also has a relatively high flow rate compared to other pumps, making it superior in terms of the rate of heat transfer ($H=Q\rho C\Delta T$), where Q: flow rate, ρ: liquid density, C: specific heat of the liquid, ΔT: temperature difference.

Reference	Pumping principle	Max flow rate [ml/min]	Typical size [cm^3]	Application
This study	Electrohydrodynamics	31	21	Pocketable thermal control
[20]	Electrohydrodynamics	6	1.17	Soft robotic, Wearable thermal control
[21]	Electrohydrodynamics	52	7.22	Soft robotic, Wearable thermal control
[22]	Magnetohydrodynamics	325	23	Soft robotic
[23]	Piezoelectric	1.1	1.98	Biological
[24]	Electromagnetic	9.5	3.5	Medical
[25]	Electroosmotic	0.001	0.01	Medical
[26]	Ionic	0.005	0.8	Micro robotic
[27]	Pneumatic	0.09	6	Micro fluidic
[28]	Electro-pneumatic	161	17	Soft robotic
[29]	Thermo-mechanical	54	80.4	Soft robotic
[30]	Electromagnetic	500	58.6	Dosing, Medical device, Cooling system, Lubrication
[31]	Electromagnetic	42500	75500	N/A
[32]	Diaphragm	400	5.85	Beauty apparatus, Air sampler, Blood pressure meter
[40]	Piezoelectric	3.12	27.2	N/A

Table S1. Pump operating principle, maximum flow rate, size, and application are summarized.

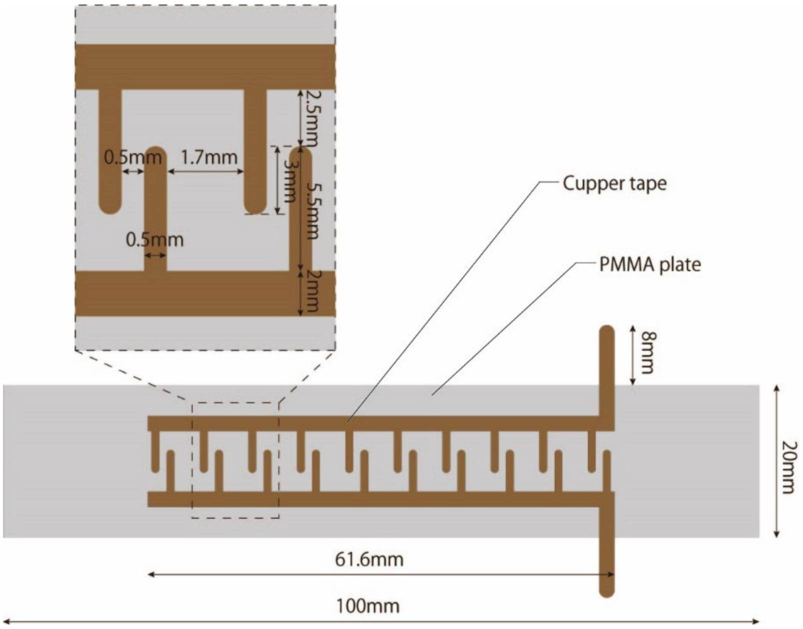

Figure S2. Electrode design of PSEP.

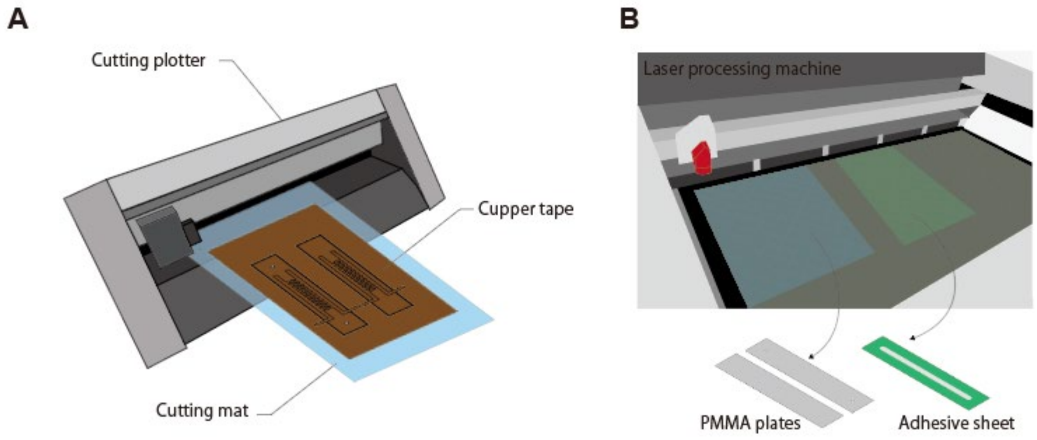

Figure S3. Digital and rapid fabrication process of **(A)** electrode, **(B)** substrate, and channel layers.

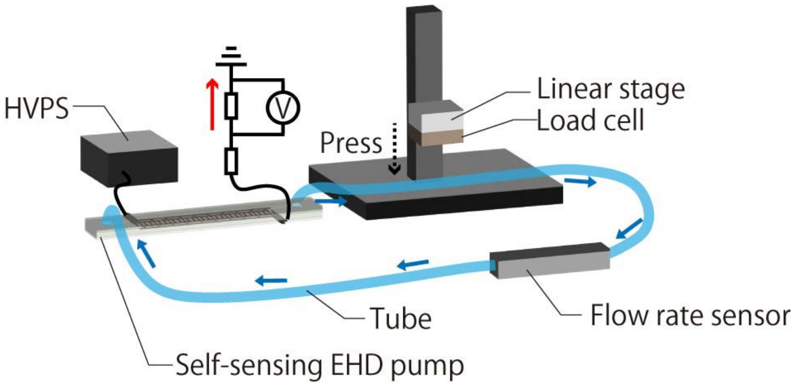

Figure S4. Experimental setup.

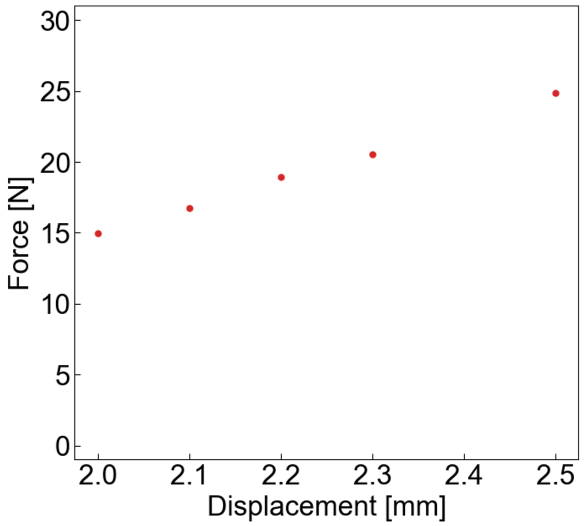

Figure S5. Compression test to clarify the relationship of tube deformation to the load applied to the tube.

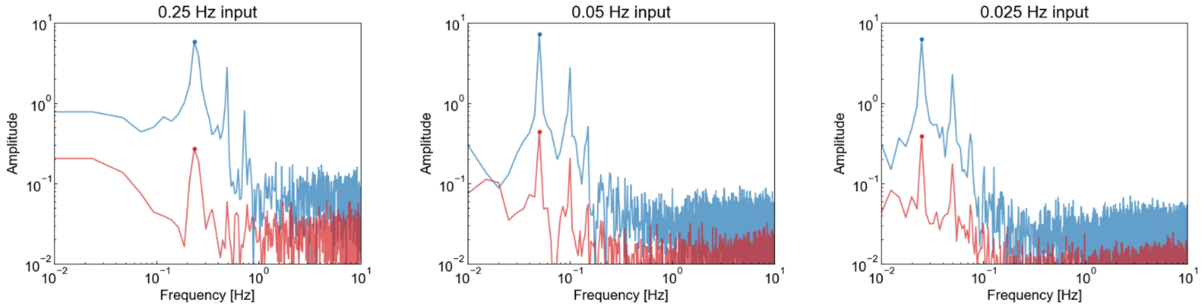

Figure S6. FFT analysis of currents and flow rates for cycling tests with left) 0.025 Hz, (middle) 0.05 Hz, and (right) 0.25 Hz frequency inputs.

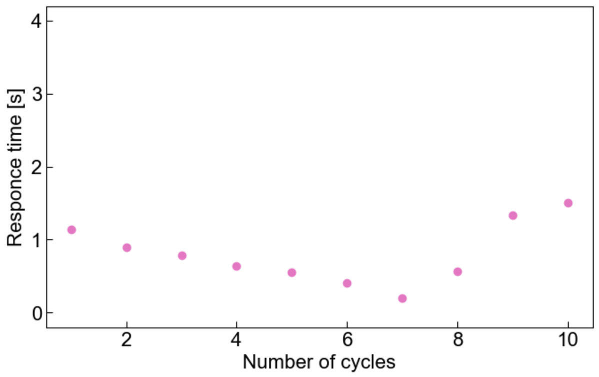

Figure S7. The response time (t_2-t_1) as the difference between the time of the lower peak of the flow rate (t_1) and that of current (t_2) at 0.05 Hz input.

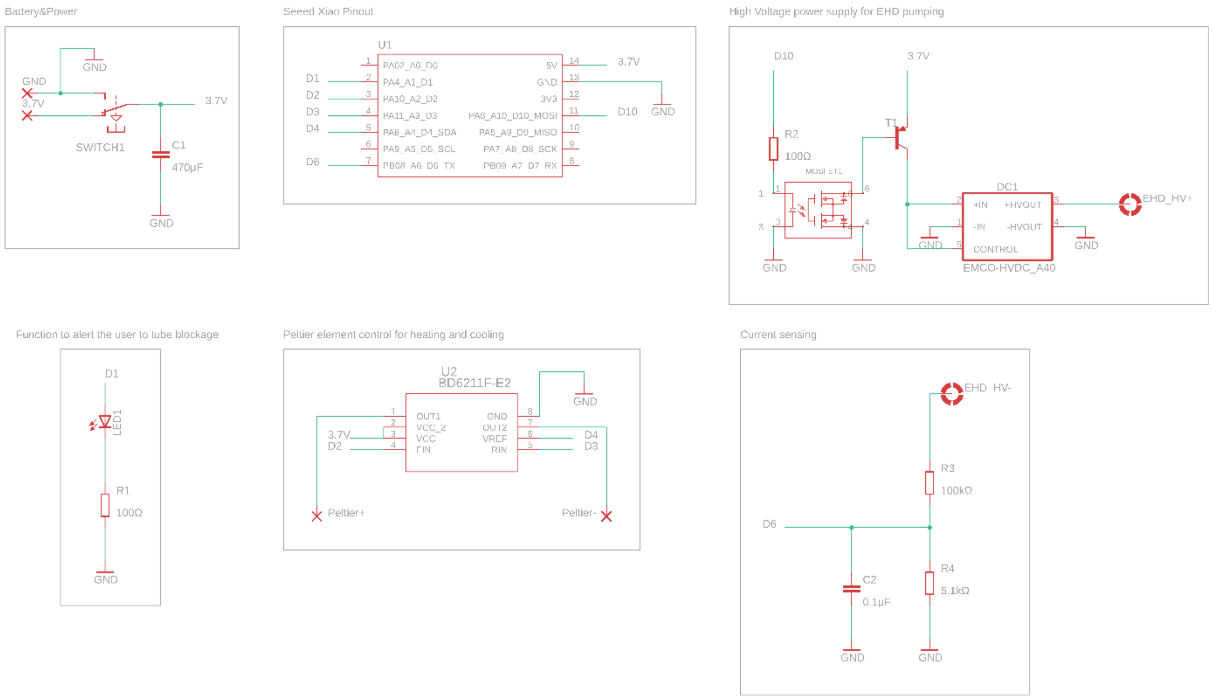

Figure S8. Electrical schematic of the power/control circuit.

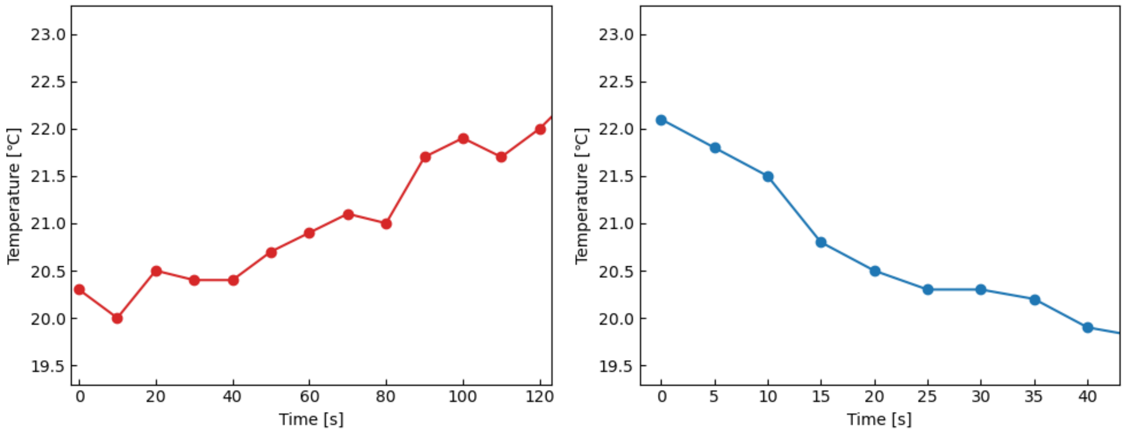

Figure S9. Temperature versus time in heating (left) and cooling (right) demonstrations.

Supplementary video description

Video S1 Heating function of wearable application.

Temperature of the tubes placed in the arms increases as the 3 kV applied PSEP transports the liquid warmed by the Peltier element. Initial (top right) and current temperature distributions (bottom right).

Video S2 Cooling function of wearable application.

Temperature of the tubes placed in the arms increases as the 3 kV applied PSEP transports the liquid cooled by the Peltier element. Initial (top right) and current temperature distributions (bottom right).

Video S3 Blockage detection, wireless control, and monitoring functions of wearable application.

Operation of the Peltier element and PSEP, abnormal alerts, and current decrease monitoring were performed on a smartphone.
